# Quadrature Errors and DC Offsets Calibration of Analog Complex Cross-Correlator for Interferometric Passive Millimeter-Wave Imaging Applications

**DOI:** 10.3390/s18020677

**Published:** 2018-02-24

**Authors:** Chao Wang, Xin Xin, Bingyuan Liang, Zhiping Li, Jungang Miao

**Affiliations:** School of Electronics and Information Engineering, Beihang University, Beijing 100191, China; austin423@buaa.edu.cn (C.W.); alisonxinxin@buaa.edu.cn (X.X.); liangby@buaa.edu.cn (B.L.); jmiaobremen@buaa.edu.cn (J.M.)

**Keywords:** analog correlator, cross correlation, interferometric imaging, millimeter-wave imaging, security sensing

## Abstract

The design and calibration of the cross-correlator are crucial issues for interferometric imaging systems. In this paper, an analog complex cross-correlator with output DC offsets and amplitudes calibration capability is proposed for interferometric passive millimeter-wave security sensing applications. By employing digital potentiometers in the low frequency amplification circuits of the correlator, the outputs characteristics of the correlator could be digitally controlled. A measurement system and a corresponding calibration scheme were developed in order to eliminate the output DC offsets and the quadrature amplitude error between the in-phase and the quadrature correlating subunits of the complex correlator. By using vector modulators to provide phase controllable correlated noise signals, the measurement system was capable of obtaining the output correlation circle of the correlator. When injected with −18 dBm correlated noise signals, the calibrated quadrature amplitude error was 0.041 dB and the calibrated DC offsets were under 26 mV, which was only 7.1% of the uncalibrated value. Furthermore, we also described a quadrature errors calibration algorithm in order to estimate the quadrature phase error and in order to improve the output phase accuracy of the correlator. After applying this calibration, we were able to reduce the output phase error of the correlator to 0.3°.

## 1. Introduction

Interferometric imaging is a well-known technique for earth remote sensing [1,2] and radio astronomy [3,4] and has received increasing attention in security sensing applications, such as concealed-weapons and explosives detection [5,6,7,8,9]. The basic element of the interferometric imaging system is the two-element interferometric correlation radiometer [9] which consists of two receiver chains (an antenna and a receiver) and a cross-correlator. The basic measurement of the interferometric correlation radiometer is the complex cross-correlation between the outputs of the two receiver chains [10]. Each complex cross-correlation measurement via the interferometric imaging system can be named as a sample of the visibility function [11]. Based on the van Cittert–Zernike theorem [12], the radiometric temperature distribution in the field of view is equivalent to the inverse Fourier transform of the visibility function samples [9,13]. Since the cross-correlator is used to implement visibility function measurement [9,14,15,16,17,18] in the interferometric imaging systems, and since the image of the source of interest is derived by using the measured visibility [10], the cross-correlator is a vital component and the quality of the image is significantly affected by the output characteristics of the cross-correlator.

Due to a limited observation time [9] and high radiometric sensitivity requirements, a broad correlation bandwidth is preferred [15] for interferometric passive millimeter-wave imagers used for security sensing applications. In practice, both digital and analog correlators can be used to implement cross-correlation measurements. In comparison, analog correlators are more suitable for high brightness sensitivity observations due to their large bandwidth and low cost [14,15,16,19,20,21,22]. A digital correlator with a 180 MHz bandwidth has been adopted in our prototype security scanner [13,23], but in order to increase the imaging sensitivity at a relatively low cost, an analog complex cross-correlator operating over 1.5–2.5 GHz was proposed in our previous publication [15]. However, for practical analog complex correlators, the quadrature amplitude error and the quadrature phase error will degrade the sensitivity of the correlator and lead to the distortion of the measured visibility function; additionally, the outputs DC offsets would also cause offsets in the measured visibility and would influence the outputs dynamic range of the correlator [20,21,24]. These problems also exist in digital systems. Therefore, in order to minimize the non-ideal behavior of the analog correlator and achieve an accurate visibility measurement, an appropriate circuit structure should be selected. Meanwhile, suitable calibration schemes are also required in order to correct the correlator’s outputs.

It is generally difficult to tune the RF characteristic of the analog correlator in the passband after it is manufactured. By assuming that the best possible RF performance is achieved for an analog complex correlator, a feasible way of reducing the amplitude errors and the DC offsets is to tune the readout circuit of the correlator. Meanwhile, in order to make better use of the quantization bits of the analog to digital converter (ADC) that follows the analog correlator, the output dynamic range of the correlator should approximate the ADC's input range. Since the power of the RF signals injected into the correlator may vary, the gain and the offsets of the readout circuit should be adjustable so as to keep the output of the correlator well-matched with the input range of the ADC [20]. In [14,15,16,19,24], the gain and the DC offsets of the readout circuits were fixed and could not be conveniently adjusted if the application circumstance was changed. Moreover, for an imaging system which requires multichannel correlators, the consistency of amplitudes and DC offsets between channels should be maintained, and this would require a hardware calibratable circuit structure for the correlator. Therefore, an analog complex correlator capable of adjusting the outputs amplitudes and DC offsets would be preferred in a real application environment. However, the quadrature phase error which is caused by the non-ideal characteristic of the RF circuit cannot be cancelled by tuning the readout circuit. Consequently, a calibration algorithm aimed at eliminating the quadrature errors should be developed. The measured visibility can be calibrated if the quadrature amplitude and phase errors of the complex correlator are known [21]. By applying an exactly 90° phase change to one of the receiver local oscillators (LO) in the interferometric imager, the quadrature errors of the complex correlator can be obtained, after which the measured cross-correlation is calibrated [24]. The quadrature errors could be efficiently estimated by this approach; however, the performance of this method relies on the phase shift accuracy of the local oscillator; the output DC offsets of the complex correlator are also neglected, which would influence the results of the calibrated cross-correlation.

In this paper, the structure of an output amplitudes and DC offsets digital tunable analog complex cross-correlator for interferometric passive millimeter-wave imaging applications is proposed. By adopting digital potentiometers in the readout circuit of the correlator, the output characteristics could be quickly and conveniently adjusted. A 1.5–2.5 GHz analog complex cross-correlator employing the proposed readout circuit structure is measured. Meanwhile, a measurement system which adopts vector modulators to control the phase of the local oscillators in a single baseline interferometer is developed to evaluate the output DC offsets and amplitudes of the correlator. Following this, the best achievable output characteristics of the correlator could be obtained by tuning the digital potentiometers. Furthermore, by developing a calibration approach which applies a 0–360° phase sweep to one of the receiver local oscillators in the interferometer, the residual DC offsets and the averaged quadrature amplitude and phase errors of the correlator can be obtained.

In Section 2, the structure of the output digital tunable analog complex cross-correlator is described. The hardware calibration scheme and the quadrature errors calibration algorithm are introduced in Section 3. The experimental results of a practical hardware calibratable correlator are presented in Section 4, and the paper is concluded in Section 5.

## 2. Correlator Architecture

### 2.1. Diode-Based Analog Complex Cross-Correlator

**Figure 1:**
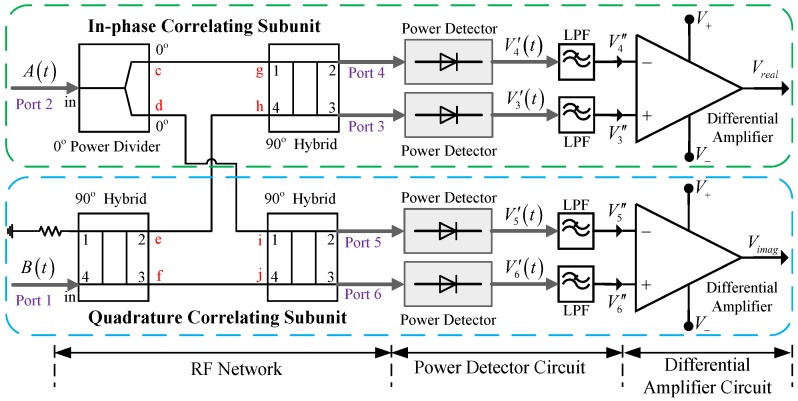
A block diagram of the diode-based analog complex cross-correlator.

Based on different nonlinear elements, analog correlators can be implemented by mixers or multipliers [14,16,20,21,24] and by diodes [15,19,25,26,27]. Due to the high stability, large bandwidth, and widespread component availability of a diode, a diode-based analog correlator is adopted here. The architecture of the correlator is shown in Figure 1 [28]. Assuming the inputs of the correlator are presented as

(1) A(t)=acos(ωt)

(2) B(t)=bcos(ωt+ϕ)

the output of the correlator can then be formulated as

(3) $$V_{real}=abG_{real}\cos\left(\phi \right)+C_{real}$$

(4) $$V_{imag}=abG_{imag}\sin\left(\phi \right)+C_{imag}$$

where $G_{*}$ ($G_{real}$ and $G_{imag}$) and $C_{*}$ ($C_{real}$ and $C_{imag}$) are the gain and the output DC offset of the low frequency differential amplifier, respectively. The detailed working principle of the correlator is described in [15].

Ideally, if $G_{real}=G_{imag}$ and $C_{real}=C_{imag}=0$, the phase and amplitude of the cross-correlation function can be evaluated precisely from the outputs of the correlator. In practice, due to the non-ideal behavior of the actual devices, the gain of the two differential amplifiers cannot be identical, and their output DC offsets would be nonzero. Thus calibration is needed in order to prevent the degradation of the correlator's performance.

### 2.2. Digital Tunable Readout Electronics

Generally, the gain of the low frequency amplification circuit in the analog correlator is determined by both the input signals and the input voltage range of the ADC that follows the correlator. In practice, the amplification value of the low frequency amplifier is set by fixed resistors [14,16,29]. Therefore, the fixed resistors must be replaced if amplitude tuning is required. In [15,16,29], the DC offset of the correlator output signal can be tuned by using potentiometers. However, if a mechanical potentiometer is adopted, it cannot be easily adjusted after the correlator is housed in a mechanical box. As a consequence, an appropriate amplification circuit structure able to conveniently adjust the amplitude and DC offset of the output signal should be developed.

**Figure 2:**
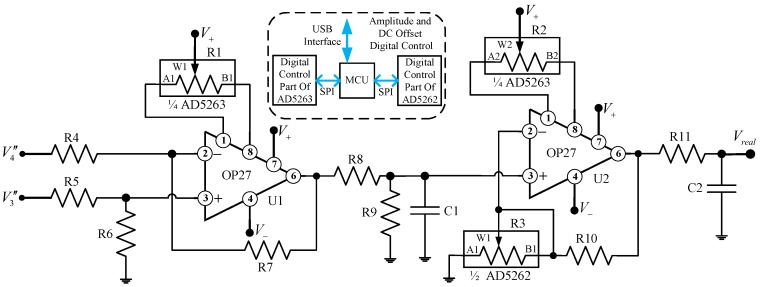
The schematic of the digital programmable low frequency amplification circuit of the in-phase correlating subunit.

The schematic of the proposed digital programmable low frequency amplification circuit is presented in Figure 2. The core devices of the circuit are the digital potentiometers (AD5263 and AD5262 from Analog Devices (Norwood, MA, USA)) which are capable of digitally tuning the resistance between the terminal (A or B) and the wiper (W). Compared with a mechanical potentiometer, a digital potentiometer has a more compact physical size, a faster adjustment capability, and a higher resolution and reliability [30]. In Figure 2, the amplitude of the output signal can be adjusted by tuning R3, while coarse and fine output DC offset adjusting is conducted by tuning R1 and R2, respectively. The control process of the digital potentiometers is as follows: the microcontroller unit (MCU) receives the control instruction from the industrial control computer (IPC) via USB interface. Then, according to the control instruction, the MCU tunes the digital potentiometer chips through the SPI interface.

By using the proposed circuit structure, a fast calibration capability of the outputs amplitudes and DC offsets of the analog complex correlator can be achieved. Meanwhile, this circuit is well suited for applications that require a number of high consistency analog correlators.

## 3. Calibration of the Correlator

### 3.1. Hardware Calibration Scheme

**Figure 3:**
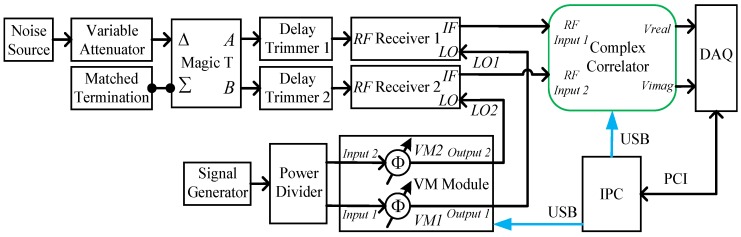
The correlator’s outputs DC offsets and quadrature errors calibration measurement test bench.

From the analysis in Section 2.1, we know that the correlator should be calibrated in order to implement the cross-correlation measurement accurately. We developed a calibration system, as illustrated in Figure 3. The noise source and the attenuator are used to provide a power adjustable correlated noise signal for the system. It should be noted that the correlated noise calibration standards (CNCS) developed in [31] could also be adopted if the statistical property of the noise signal needs to be adjustable. The magic T serves as a power divider for the noise signal, and the delay trimmers are used to tune the time delay between the RF paths. The vector modulator (VM) module [32] is employed to control the phase of the local oscillator signals for the receivers. We assume that the outputs of the signal generator and the VMs are

(5) $$V_{SG}(t)=A_{LO}\cos\left(\omega_{LO}t\right)$$

(6) $$V_{VM1}(t)=A_{LO}\cos\left[\omega_{LO}t+\phi_{LO}\right]$$

(7) $$\begin{array}{cc} \phi_{LO}=\frac{2\pi \left(n-1\right)}{N} & \frac{nT}{N}-\frac{T}{N}<t<\frac{nT}{N} \end{array}$$

(8) $$V_{VM2}(t)=A_{LO}\cos\left(\omega_{LO}t\right)$$

$\phi_{LO}$ is the phase difference between the LOs of the two receivers. *T* is the period VM1 needs to sweep the phase of $V_{VM1}(t)$ between 0–360°. *N* is the total number of phase states in the 0–360° phase sweep, and *n* is the *n*th phase state, n=1,2,⋯N. From the expression of $V_{VM1}(t)$, it experiences a 0–360° phase sweep during the period of time *T*, and there are *N* phase states in the phase sweep. The phase of $V_{VM2}(t)$ is fixed, but a 180° phase-switching scheme [14,19,21,24,33] can also be employed in VM2, which would enable the removal of the offset drift [33] and the cross-talk presented in the imaging system [19].

The two receivers and the complex correlator in Figure 3 can be regarded as a single baseline interferometer; for an interferometer baseline which uses a complex correlator with a rectangular passband of radian bandwidth *B*, the outputs are [19,34]

(9) $$R=R_{o}\mathrm{sinc}\left[B\left(\tau_{g}-\tau_{i}\right)\right]\cos\left\{2\pi \left[\omega_{LO}\tau_{g}-\omega_{IF}\left(\tau_{g}-\tau_{i}\right)\right]+\phi_{LO}\right\}$$

(10) $$I=R_{o}\mathrm{sinc}\left[B\left(\tau_{g}-\tau_{i}\right)\right]\sin\left\{2\pi \left[\omega_{LO}\tau_{g}-\omega_{IF}\left(\tau_{g}-\tau_{i}\right)\right]+\phi_{LO}\right\}$$

where $R_{o}$ is the normalization parameter, $\tau_{g}$ and $\tau_{i}$ are the time delay presented at RF and IF between the two receivers, respectively, $\omega_{LO}$ and $\omega_{IF}$ are the LO and IF radian frequency, respectively.

In our calibration system, $\tau_{i}$ is small due to the high level of symmetry in the IF path, and $\tau_{g}$ is tunable, while $\phi_{LO}$ can be adjusted by using the VMs. Following this, the outputs of the correlator in the calibration system can be formulated as

(11) $$V_{real}\left(\phi_{LO}\right)=G_{real}\mathrm{sinc}\left[B\left(\tau_{g}-\tau_{i}\right)\right]\cos\left\{2\pi \left[\omega_{LO}\tau_{g}-\omega_{IF}\left(\tau_{g}-\tau_{i}\right)\right]+\phi_{LO}\right\}+C_{real}$$

(12) $$V_{imag}\left(\phi_{LO}\right)=G_{imag}\mathrm{sinc}\left[B\left(\tau_{g}-\tau_{i}\right)\right]\sin\left\{2\pi \left[\omega_{LO}\tau_{g}-\omega_{IF}\left(\tau_{g}-\tau_{i}\right)\right]+\phi_{LO}\right\}+C_{imag}$$

(13) $$\phi_{LO}=\frac{2\pi \left(n-1\right)}{N}$$

**Figure 4:**
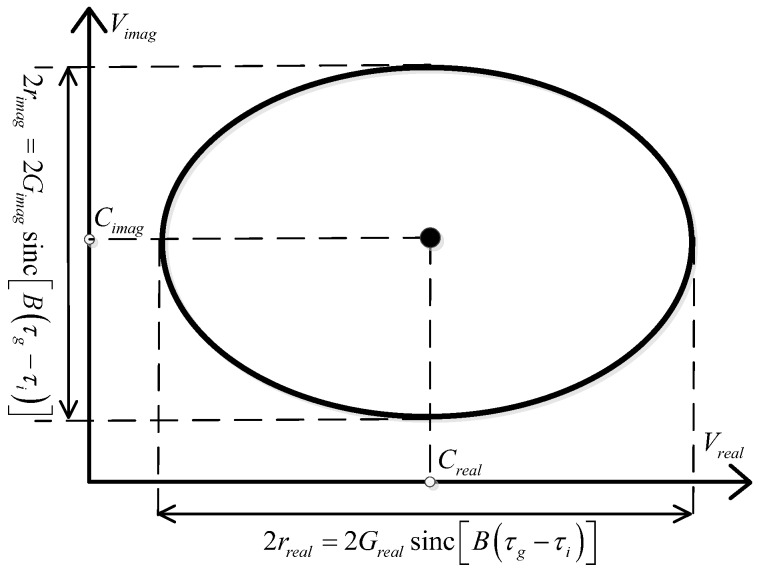
The output correlation circle of a complex correlator.

It can be seen that, due to the phase sweep of LO1, the outputs of the complex correlator depend on the phase difference between VM1 and VM2. By sweeping $\phi_{LO}$ from 0–360°, $V_{real}\left(\phi_{LO}\right)$ and $V_{imag}\left(\phi_{LO}\right)$ can be plotted on a polar diagram. This curve is named as a correlation circle and is shown in Figure 4. The outputs of the complex correlator describe an ellipse. The center of the ellipse is the DC offset presented in the in-phase and quadrature correlating subunit of the complex correlator. The major axis ($2r_{real}=2G_{real}\mathrm{sinc}\left[B\left(\tau_{g}-\tau_{i}\right)\right]$) and the minor axis ($2r_{imag}=2G_{imag}\mathrm{sinc}\left[B\left(\tau_{g}-\tau_{i}\right)\right]$) of the ellipse are proportional to the gain of the low frequency amplification circuit in the in-phase and quadrature correlating subunit, respectively. Meanwhile, the axial ratio ($AR=\frac{r_{real}}{r_{imag}}$) reflects the amplitude imbalance between the in-phase and quadrature correlating subunit, and the closer the axial ratio is to 1, the better the amplitude symmetry is.

**Figure 5:**
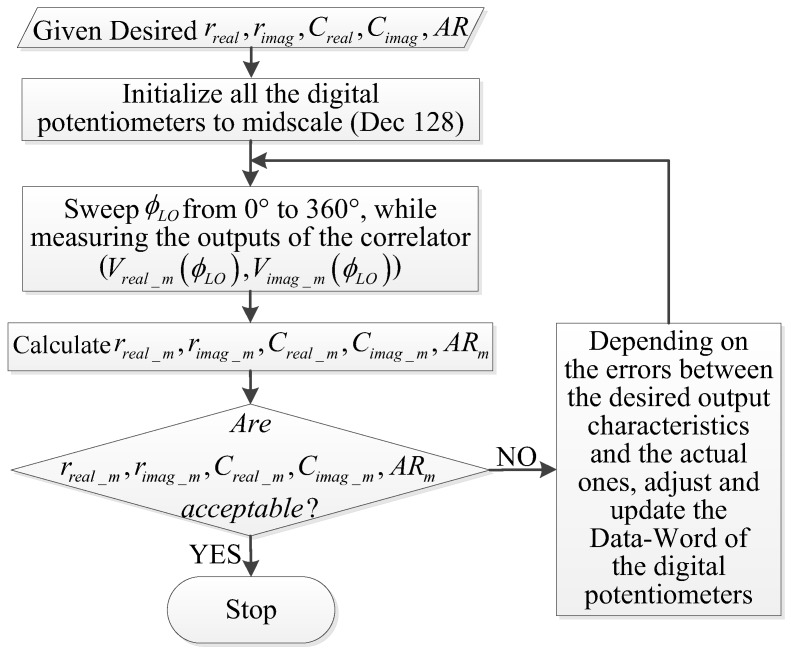
The flowchart for the DC offsets and the quadrature amplitude calibration.

Based on the above descriptions, by sweeping the phase difference between the LOs of the receivers while measuring the outputs of the complex correlator, the outputs amplitudes and DC offsets of the correlator can be determined. Subsequently, the correlator is able to be calibrated by tuning the digital potentiometers in the low frequency amplification circuit. The flow chart of the calibration procedure is illustrated in Figure 5.

It can be noted that after a 0–360° phase sweep is finished for $\phi_{LO}$, the corresponding measured $V_{real\_m}\left(\phi_{LO}\right)$ and $V_{imag\_m}\left(\phi_{LO}\right)$ can be used to calculate the actual outputs characteristics ($r_{real\_m},r_{imag\_m},C_{real\_m},C_{imag\_m},AR_{m}$) of the complex correlator, which are formulated as

(14) $$r_{real\_m}=\frac{\max\left[V_{real\_m}\left(\phi_{LO}\right)\right]-\min\left[V_{real\_m}\left(\phi_{LO}\right)\right]}{2}$$

(15) $$r_{imag\_m}=\frac{\max\left[V_{imag\_m}\left(\phi_{LO}\right)\right]-\min\left[V_{imag\_m}\left(\phi_{LO}\right)\right]}{2}$$

(16) $$C_{real\_m}=mean\left[V_{real\_m}\left(\phi_{LO}\right)\right]$$

(17) $$C_{imag\_m}=mean\left[V_{imag\_m}\left(\phi_{LO}\right)\right]$$

(18) $$AR_{m}=\frac{r_{real\_m}}{r_{imag\_m}}$$

Following this, whether one increases or decreases the resistance values of the digital potentiometers and the corresponding adjusting step size depends on the differences between the desired outputs characteristics and the actual ones. The calibration process is finished when the output characteristics of the complex correlator are acceptable. It should be noted that the best achievable outputs characteristics could be obtained when the tolerances for the desired outputs characteristics are extremely small. Finally, the Data-Word (which is the data that is used to set the resistance value of the digital potentiometer) are stored.

### 3.2. Quadrature Errors and Residual DC Offsets Calibration Algorithm

The calibration scheme presented above is a hardware-based calibration method, and the best achievable outputs characteristics of the complex correlator rely on the resolution of the digital potentiometers. Due to finite resolution, the amplitude imbalance and the residual DC offsets may still exist, but they are very small. However, the quadrature phase error caused by the non-ideal behavior of the RF circuit cannot be cancelled by the hardware-based calibration scheme.

For a non-ideal complex correlator, we assume that the amplitude of the quadrature correlating subunit is g times that of the in-phase correlating subunit and that the quadrature phase error is ε. The quadrature errors (g and ε) can be determined and further calibrated by applying an exactly 90° phase change to the LO of one of the receivers [24]. This is an efficient way to calibrate the quadrature errors, but the DC offsets of the correlator are neglected and this approach requires a relatively high phase shift accuracy for the LO. To improve the reliability and accuracy for the quadrature errors calibration, a calibration algorithm based on a LO phase shift scheme is developed, capable of obtaining the averaged quadrature errors and less demanding for the phase shift accuracy of the LO. Furthermore, the residual DC offsets following the hardware calibration are also considered in this algorithm. The procedure for this calibration algorithm is as follows. Firstly, we assume that the phase states of LO1 and LO2 in Figure 3 are $\phi_{LO}$ and 0°, respectively; $\tau_{g}=\tau_{i}=0$, and $\rho =ae^{i\theta }$ is the actual cross-correlation of the correlated signals injected into the receivers. Therefore, the output of the non-ideal complex correlator is

(19) $$r(\phi_{LO})=a\cos(\theta +\phi_{LO})+C_{real}+i\left[ag\sin(\theta +\phi_{LO}+\varepsilon )+C_{imag}\right]$$

Following this, the phase is shifted by approximately 90° for LO1. It should be noted that if the VM is adopted and calibrated to control the phase state of the LOs, the phase shift error can be less than 1°. The detailed calibration process for the VM can be seen in our previous work [32].

Subsequently, the output of the non-ideal complex correlator would be

(20) $$r_{q}(\phi_{LO})=-a\sin\left(\theta +\phi_{LO}\right)+C_{real}+i\left[ag\cos\left(\theta +\phi_{LO}+\varepsilon \right)+C_{imag}\right]$$

$r_{q}(\phi_{LO})$ is formulated under the assumption that the phase state of LO1 is changed from $\phi_{LO}$ to $\phi_{LO}+90^{{}^{\circ}}$.

From [24], the quadrature errors could be determined as

(21) ε(ϕLO)=arctan[Im[r(ϕLO)−iCimag]Im[rq(ϕLO)−iCimag]]−arctan[−Re[rq(ϕLO)−Creal]Re[r(ϕLO)−Creal]]

(22) g(ϕLO)=[Im[r(ϕLO)−iCimag]]2+[Im[rq(ϕLO)−iCimag]]2[Re[r(ϕLO)−Creal]]2+[Re[rq(ϕLO)−Creal]]2

Theoretically, g and ε are the inherent property of the complex correlator that are decided by the hardware itself. Due to $\phi_{LO}+90^{{}^{\circ}}$ phase state cannot be exactly obtained for LO1, and there is a phase error between the desired $\phi_{LO}+90^{{}^{\circ}}$ phase state and the actual one. It should be noted that this phase error is a function of the desired phase state, since the phase shift accuracy of the VM varies with the desired phase state. For instance, the phase errors may be 0.8° and 0.5° for the phase states at 60° and 80°, respectively. Therefore, the phase shift error of the VM is included in $\varepsilon (\phi_{LO})$ and $g(\phi_{LO})$.

In practice, by assuming that an exactly 90° phase change is obtained for LO1 and that the DC offsets ($C_{real}$, $C_{imag}$) are equal to zero, $\varepsilon (\phi_{LO})$ and $g(\phi_{LO})$ can be calculated by using Equation (21) and (22). Indeed, due to the non-exact 90° phase change and the nonzero DC offsets, there would be some deviations between the estimated quadrature errors ($g(\phi_{LO})$ and $\varepsilon (\phi_{LO})$) and the actual ones (g and ε). However, these deviations usually being small, $g(\phi_{LO})$ and $\varepsilon (\phi_{LO})$ could be used to estimate g and ε, respectively. Since the estimated quadrature errors are dependent on $\phi_{LO}$, $g(\phi_{LO})$ and $\varepsilon (\phi_{LO})$ averaged over $\phi_{LO}$ ($\bar{g}$ and $\bar{\varepsilon }$) would be more suitable for determining g and ε. $\bar{g}$ and $\bar{\varepsilon }$ are formulated as

(23) $$\bar{g}=\bar{g(\phi_{LO})}=\frac{1}{2\pi }\int_{0}^{2\pi }g(\phi_{LO})d\phi_{LO}$$

(24) $$\bar{\varepsilon }=\bar{\varepsilon (\phi_{LO})}=\frac{1}{2\pi }\int_{0}^{2\pi }\varepsilon (\phi_{LO})d\phi_{LO}$$

As discussed in Section 3.1, the DC offsets of the complex correlator could be estimated via the outputs of the correlator after a 0–360° phase sweep is adopted for $\phi_{LO}$. Subsequently, the function of $g(\phi_{LO})$ and $\varepsilon (\phi_{LO})$ can be obtained, after which $\bar{g}$ and $\bar{\varepsilon }$ are determined.

After adopting the calibration algorithm, the actual cross-correlation obtained is

(25) ρ=Re[r(0)−Creal]+i[Im[r(0)−iCimag]g¯−Re[r(0)−Creal]sinε¯]1cosε¯

Furthermore, in order to investigate the performance of the quadrature errors calibration algorithm, the output phase error [15] of the complex correlator is introduced. The output phase error is formulated as

(26) $$\phi_{error}(\phi )=\arctan(\frac{V_{imag}}{V_{real}})-\phi$$

where ϕ is the phase of the cross-correlation between two correlated signals injected into the complex correlator.

Given that the outputs ($V_{real}$ and $V_{imag}$) of the complex correlator are able to reconstruct the cross-correlation phase, the output phase error can subsequently be used to estimate the phase reconstruction performance of the complex correlator. For an actual complex correlator, the phase reconstruction accuracy is affected by DC offsets and quadrature errors. Therefore, if these aspects are known, the phase reconstruction accuracy could be improved. Meanwhile, the output phase error can also be calibrated and decreased. Considering the DC offsets and the quadrature errors, the calibrated output phase error can be written as

(27) $$\phi_{error\_cal}(\phi )=\arctan\left[\frac{V_{imag}-C_{imag}}{\bar{g}\left(V_{real}-C_{real}\right)\cos\bar{\varepsilon }}-\tan\bar{\varepsilon }\right]-\phi$$

Since the DC offsets and the quadrature amplitude error could be reduced significantly by using the proposed hardware calibration, the output phase error is usually dominated by quadrature phase errors.

## 4. Experimental Realization

### 4.1. Experimental Analog Complex Cross-Correlator

**Figure 6:**
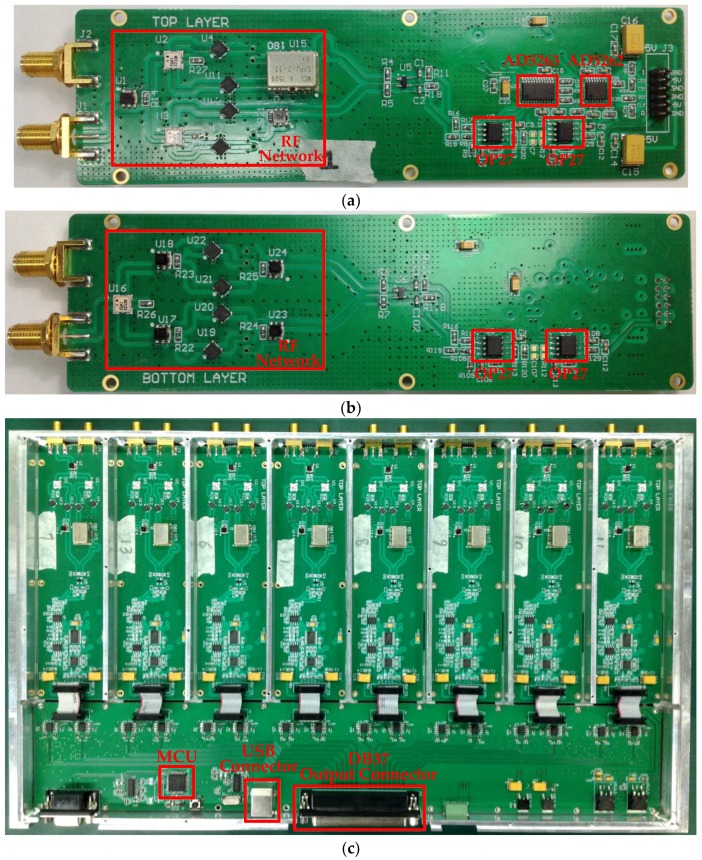
Photographs of the experimental analog complex cross-correlator: (**a**) Top layer of a correlator board; (**b**) bottom layer of a correlator board; and (**c**) eight-channel correlator module.

A diode-based analog complex cross-correlator which adopts the proposed digital programmable amplification circuit is implemented and shown in Figure 6. The top and bottom layers of the correlator are shown in Figure 6a,b, respectively. The digital potentiometer chips AD5263 and AD5262 are located in the middle of the PCB, while the operational amplifiers (OP27) of the in-phase and quadrature correlating subunit are placed on the two sides of the digital potentiometer chips. This kind of configuration aims to equalize the physical path between the amplifiers and the digital potentiometers. The function of the RF network of the fabricated correlator is identical with that of the RF network presented in Figure 1; a detailed description of the RF network in Figure 6a,b is presented in our previous work [15]. As the correlator is designed for an imaging system which requires 128 correlators, an 8 channel complex correlator module is designed and shown in Figure 6c. The MCU is used to communicate with the IPC and to manipulate the digital potentiometer chips on the eight correlators, and the outputs of the eight correlators can be accessed from the DB37 connector.

### 4.2. Experimental System

**Figure 7:**
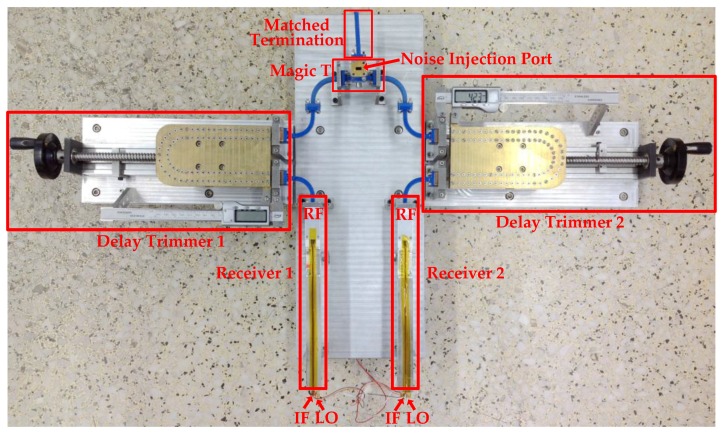
Photograph of the main parts in the experimental system.

The experimental system is configured according to the schematic in Figure 3. Figure 7 shows the photograph of the main parts in the actual experimental system. The specific parameters of the main modules in Figure 3 and Figure 7 are given in Table 1. The RF delay trimmer in Figure 7 can be adjusted by tuning the manual handle. A multichannel VM module developed in [32] is used to control the LO phase. It should be noted that the output frequency of the VM module is 2 GHz, which is then frequency multiplied to 32 GHz for the LO of the receiver.

### 4.3. Hardware Calibration of the Outputs Amplitudes and DC Offsets

This measurement aims to calibrate the correlator outputs amplitudes and DC offsets by tuning the digital potentiometers. Before implementing this test, the variable attenuator should be adjusted in order to achieve the desired input signal power for the correlator.

**Figure 8:**
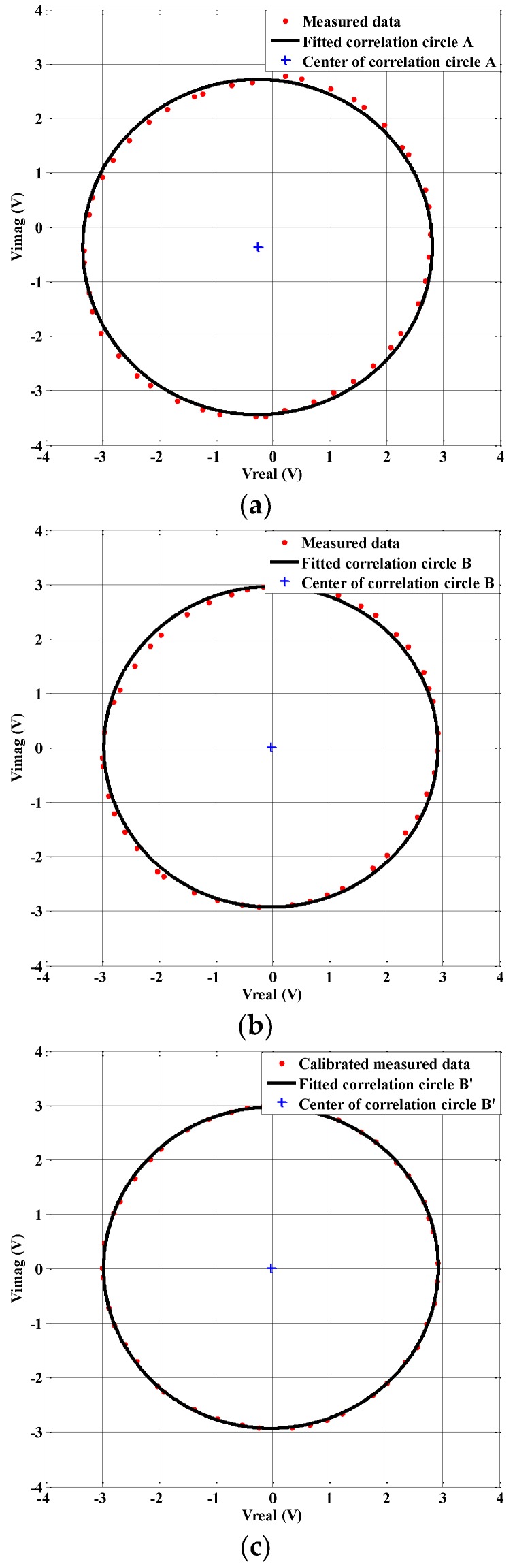
(**a**) The measured output correlation circle without hardware calibration; (**b**) the measured output correlation circle with hardware calibration; and (**c**) the output correlation circle after the quadrature errors calibration.

The experiment is accomplished according to the process described in Section 3.1. By sweeping $\phi_{LO}$, a set of the outputs of the complex correlator ($V_{real\_m}\left(\phi_{LO}\right)$ and $V_{imag\_m}\left(\phi_{LO}\right)$) are obtained. Following this, the measured data is adopted to fit the correlation circles in Figure 8a,b. Circle A corresponds to the hardware uncalibrated correlation circle, while circle B corresponds to the correlation circle following the hardware calibration. The specifications of these circles are listed in Table 2. In our applications, the correlation circle origin offsets must be less than 30 mV, the axial ratio should be within the range of 0.98–1.02, while the radius of the correlation circle should be within 2.9–3.1 V when the input signal power of the correlator is approximately −18 dBm. As shown in Table 2, the DC offsets of circle A are too large due to the uncalibrated digital potentiometers R1 and R2 in Figure 2. The axial ratio of circle A is also unacceptable due to the fact that the gain of the operational amplifier U2 (Figure 2) is relatively low in the in-phase correlating subunit. By tuning the digital potentiometer R3, shown in Figure 2, the axial ratio could be calibrated. After hardware calibration, the origin offsets of correlation circle B are less than 26 mV, which is only 7.1% of the hardware uncalibrated correlation circle A, while the axial ratio, the circle radius, and the RMS fitting error are 1.0047, 2.942 V, and 0.02383, respectively, all of which are acceptable for our application. The correlation circle RMS fitting error indicates the deviation between the measured data and the fitted correlation circle. A smaller RMS fitting error reveals a better complex cross-correlation performance. From correlation circles A and B, it can be concluded that by tuning the digital potentiometers in the low frequency amplification circuits of the analog complex correlator, the outputs characteristics of the correlator can be adjusted and calibrated.

After the hardware calibration, a look-up table storing the Data-Word of the calibrated digital potentiometers can be made. If the correlator is intended to be used in several different circumstances, different outputs characteristics would be required for the same correlator. By loading a different Data-Word, the outputs characteristics of the correlator could be quickly and conveniently configured, which is preferred in practice.

### 4.4. Calibration of the Quadrature Errors and Residual DC Offsets

The DC offsets and amplitude unbalance could be minimized by the hardware calibration process. However, the quadrature errors of the analog complex correlator, in particular the quadrature phase error, cannot be eliminated via the hardware calibration. Therefore, the quadrature errors calibration algorithm described in Section 3.2 is applied to the measured data of the correlation circle B in Figure 8b. Figure 8c plots the measured data after the quadrature errors calibration, as well as the corresponding fitted correlation circle B’. Compared with Figure 8b, it is evident that the calibrated measured data in Figure 8c are closer to the fitted correlation circle B’. The specifications of circle B’ are also summarized in Table 2. When comparing circle B’ with B, one can see that the axial ratio of B’ is closer to 1. This is because the quadrature errors are calibrated. The correlation circle RMS fitting error of circle B’ is much less than B, which indicates that B’ more closely approximates the ideal correlation circle.

**Figure 9:**
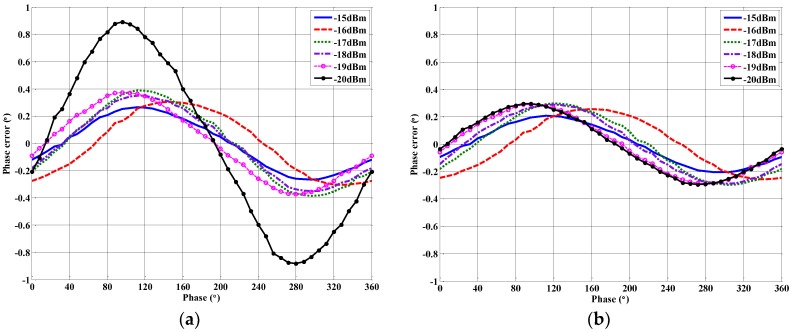
(**a**) The uncalibrated phase error at different input power levels; and (**b**) the calibrated phase error at different input power levels.

To further illustrate the performance of the quadrature errors calibration algorithm, Figure 9 plots the correlator’s output phase error at different input power levels with and without the quadrature errors calibration. It can be noted that the power levels in the experiment are decided by the correlator's final application scenario. The specifications of the uncalibrated and calibrated output phase errors are listed in Table 3. At a −20 dBm input power level, the maximum output phase error of the correlator is about 0.9°, but this could be reduced to 0.3° after the quadrature errors calibration. From Table 3, it can be concluded that the mean phase, the peak–peak phase and the RMS fitting errors can all be reduced through a quadrature errors calibration. After calibration, the maximum output phase errors for all input power levels are lower than 0.3° but higher than 0.2°, and this is mainly due to the measurement errors and the phase shift error of the VM. Generally, for most applications, the calibrated 0.3° output phase error is acceptable.

By using a hardware calibration, the DC offsets and the quadrature amplitude error of the correlation circle B are only 26 mV and 0.041 dB, respectively. It can be seen that a further reduction can be achieved if higher resolution digital potentiometers are adopted in the correlator. The 8-bit digital potentiometers are acceptable for our current correlator version. However, for demanding applications, 16-bit digital potentiometers may be employed. After applying the quadrature errors calibration algorithm, the RMS fitting error and the quadrature amplitude error of the calibrated correlation circle (B’) are reduced to 0.00163 and 0.02 dB, respectively. Meanwhile, the calibrated output phase error is lower than 0.3°. It can be concluded that the DC offsets and the quadrature amplitude error are reduced by the hardware calibration scheme, while the residual DC offsets and the quadrature errors can be further corrected after applying the quadrature errors calibration algorithm. It should additionally be noted that it is possible to avoid the hardware calibration process and directly apply the quadrature errors calibration algorithm. However, the hardware uncalibrated outputs of the complex cross-correlator may exceed the input range of the ADC that follows the correlator. In practice, it would therefore be desirable to implement hardware calibration first before using a quadrature error calibration.

## 5. Conclusions

We have presented the architecture of a hardware calibratable analog complex correlator for imaging applications. The outputs DC offsets and amplitudes of the correlator could be quickly and precisely adjusted by tuning the digital potentiometers in the low frequency amplification circuits. A hardware calibration scheme for evaluating and correcting the DC offsets and the quadrature amplitude error of the complex correlator was developed. After using this approach, the quadrature amplitude error could be reduced to 0.041 dB, and the DC offsets were only at 7.1% of the uncalibrated value. Meanwhile, we also described a calibration algorithm which could further determine and calibrate the residual DC offsets and the quadrature errors. The calibrated quadrature amplitude error and the output phase error of the correlator could be less than 0.02 dB and 0.3°, respectively. Due to the ease of implementation and high calibration accuracy, the hardware calibration scheme and the quadrature errors calibration algorithm are well suited for a variety of applications.

## Figures and Tables

**Table 1:** Specifications of the main modules in the experimental system.

Module	Parameter	Specification
Receiver	Center frequency	34 GHz
	LO frequency	32 GHz
	IF frequency	1.5–2.5 GHz
	Receiver type	Heterodyne, SSB (Ka-band)
RF Delay Trimmer	Delay range	1275 ps
	Delay increment	0.17 ps
Complex Correlator	Operating frequency range	1.5–2.5 GHz
	Output voltage range	−5~+5 V
Noise Source	Output frequency range	Ka-band
	ENR	15 dB
DAQ	Part number	PCI-1715U (Advantech)
	Description	500 kS/s, 12-bit, 32-ch Analog Input PCI Card

**Table 2:** Specifications of the correlation circles A, B, and B’.

Circle No.	A (Hardware Uncalibrated)	B (Hardware Calibrated)	B’ (Quadrature Errors Calibrated)
Correlation Circle Origin Offset (mV)	(−265.576,−363.641)	(−25.855,18.831)	(−25.567,19.312)
Correlation Circle RMS Fitting Error	0.02743	0.02383	0.00163
Circle Radius (V)	3.075	2.942	2.946
Axial Ratio	0.9751	1.0047	1.0023
Quadrature Amplitude Error (dB)	−0.22	0.041	0.02
Comments	Unacceptable DC offsets, axial ratio, and quadrature amplitude error	Acceptable for our application	Acceptable for our application

**Table 3:** Specifications for the uncalibrated and calibrated output phase errors.

Power (dBm)	−20		−19		−18		−17		−16		−15	
	Uncal	Cal	Uncal	Cal	Uncal	Cal	Uncal	Cal	Uncal	Cal	Uncal	Cal
Correlation Circle RMS Fitting Error	0.02860	0.01231	0.02424	0.00190	0.02383	0.00163	0.01912	0.00100	0.02190	0.00287	0.02060	0.00317
Mean Phase Error (°)	−0.0063	−0.0013	−0.0026	−0.0018	−0.0033	−0.0028	−0.0061	−0.0053	−0.0064	−0.0057	−0.0031	−0.0024
Peak-Peak Phase Error (°)	1.7725	0.5905	0.7471	0.5755	0.7017	0.5786	0.7738	0.5946	0.6094	0.5120	0.5314	0.4110
